# Antikinetoplastid Activity of Indolocarbazoles from *Streptomyces sanyensis*

**DOI:** 10.3390/biom10040657

**Published:** 2020-04-24

**Authors:** Luis Cartuche, Ines Sifaoui, Atteneri López-Arencibia, Carlos J. Bethencourt-Estrella, Desirée San Nicolás-Hernández, Jacob Lorenzo-Morales, José E. Piñero, Ana R. Díaz-Marrero, José J. Fernández

**Affiliations:** 1 Instituto Universitario de Bio-Orgánica Antonio González (IUBO AG), Universidad de La Laguna (ULL), Avda. Astrofísico F. Sánchez 2, 38206 La Laguna, Tenerife, Spain; 2 Departamento de Química y Ciencias Exactas, Sección Química Básica y Aplicada, Universidad Técnica Particular de Loja (UTPL), San Cayetano alto s/n, A.P. 1101608, Loja, Ecuador; 3 Instituto Universitario de Enfermedades Tropicales y Salud Pública de Canarias (IUETSPC), Departamento de Obstetricia y Ginecología, Pediatría, Medicina Preventiva y Salud Pública, Toxicología, Medicina Legal y Forense y Parasitología, Universidad de La Laguna, Avda. Astrofísico F. Sánchez s/n, 38206 La Laguna, Tenerife, Spain; 4 Departamento de Química Orgánica, Universidad de La Laguna (ULL), Avda. Astrofísico F. Sánchez, 2, 38206 La Laguna, Tenerife, Spain

**Keywords:** Indolocarbazole, kinetoplastid, *Streptomyces*, leishmanicidal, trypanocidal

## Abstract

Chagas disease and leishmaniasis are neglected tropical diseases caused by kinetoplastid parasites of *Trypanosoma* and *Leishmania* genera that affect poor and remote populations in developing countries. These parasites share similar complex life cycles and modes of infection. It has been demonstrated that the particular group of phosphorylating enzymes, protein kinases (PKs), are essential for the infective mechanisms and for parasite survival. The natural indolocarbazole staurosporine (STS, **1**) has been extensively used as a PKC inhibitor and its antiparasitic effects described. In this research, we analyze the antikinetoplastid activities of three indolocarbazole (ICZs) alkaloids of the family of staurosporine STS, **2**–**4**, and the commercial ICZs rebeccamycin (**5**), K252a (**6**), K252b (**7**), K252c (**8**), and arcyriaflavin A (**9**) in order to establish a plausive approach to the mode of action and to provide a preliminary qualitative structure–activity analysis. The most active compound was 7-oxostaurosporine (7OSTS, **2**) that showed IC_50_ values of 3.58 ± 1.10; 0.56 ± 0.06 and 1.58 ± 0.52 µM against *L. amazonensis; L. donovani* and *T. cruzi,* and a Selectivity Index (CC_50_/IC_50_) of 52 against amastigotes of *L. amazonensis* compared to the J774A.1 cell line of mouse macrophages.

## 1. Introduction

Neglected Tropical Diseases (NTDs) are a diverse group of tropical and subtropical infectious diseases with a high prevalence in low and middle-income populations that lack new, cost-effective treatments [1]. The World Health Organization, WHO, has identified 17 NTDs: dengue, rabies, trachoma, buruli ulcer, endemic treponematoses, leprosy, Chagas disease, human African trypanosomiasis (HAT), leishmaniasis, taeniasis/cysticercosis, dracunculiasis, echinococcosis, food-borne trematodiases, lymphatic filariasis, onchocerciasis, schistosomiasis, and soil-transmitted helminthiasis. Many of the NTDs are zoonotic and/or vector-borne [2].

A short group of NTDs caused by the so-called kinetoplastid parasites of increasing research interest include HAT, Chagas disease (American trypanosomiasis) and leishmaniasis. Kinetoplastids are flagellated unicellular protozoan mainly distinguished by the presence of a DNA-containing region known as ‘kinetoplast’ in their single large mitochondrion [3]. All of them share a common vector that transmits the parasite (arthropod vectors), a mammalian reservoir and a host. The diseases caused by kinetoplastid parasites are neglected by the global expenditures in research and development [4].

HAT is caused by two of the three subspecies of *Trypanosoma brucei* and occurs in sub-Saharan Africa regions populated by tsetse. HAT is fatal if left untreated. The first stage includes fever, headache, adenopathy, joint pain and pruritus, while the second stage is accompanied by severe neurological disorders that includes mental, sensory and sleep anomalies [3]. To treat HAT, five drugs have been approved: pentamidine, melarsoprol, eflornithine, suramin, and nifurtimox [4].

Chagas diseases is caused by *Trypanosoma cruzi* and is widely spread in Central and South America. The infection promotes heart failure, ventricular arrhythmias, heart blocks, thromboembolic phenomena, and sudden death. The disease is also observed in the south of United States and some regions of Europe due to the migratory movements of people from endemic zones [5]. The first line of treatment includes benznidazole and nifurtimox for one or two months of therapy for the acute infection and there is no effective treatment for chronic infection [6]. In a recent study, a benznidazole and posaconazole combination treatment was tested but it demonstrated to be ineffective in long-term asymptomatic *T. cruzi* carriers, whereas benznidazole monotherapy proved to be more effective [7].

Leishmaniasis is caused by more than 20 species, with the most frequent being *Leishmania amazonensis, L. tropica, L. donovani, L. infantum, L. braziliensis* and *L. mexicana.* It is widely spread across the world and the three predominant clinical manifestations are the cutaneous (CL), mucocutaneous (MC) and visceral leshmanisis (VL). The treatment is mainly based on long-period administration of pentavalent antimonial compounds (PAC) and, as a second line treatment when PAC fails, the use of AmBisome^®^ is highly recommended for VL [8].

It has been demonstrated that the particular group of phosphorylating enzymes, the protein kinases (PKs), are essential for parasite survival and for infective mechanisms, for which all three parasites share similarities. For this reason, increasing attention is given to PKs as druggable targets [8,9]. Knowledge of the kinome of *T. brucei* and *T. cruzi* has revealed crucial differences from their mammal counterparts. The trypanosomatids lack members of the receptor-linked (TK) or cytosolic tyrosine kinase families (TKL) [10]. Moreover, for *L. mexicana*, protein kinase CRK3 has been identified as essential for cell cycle progression [11] and CDK 12 as a drug target for VL [12]. Many research programs have been conducted to search for specific kinase inhibitors and, in the course of this action, several classes of compounds have been identified [9,13,14]. 

Staurosporine (STS, **1**) is a potent PKC inhibitor and has demonstrated the induction of programmed cell death (PCD) or apoptotic-like activity on parasites by several mechanisms [9,15,16,17] including protein kinase interaction or mitochondrial-related mechanisms [18]. Against *L. donovani*, STS was determined to promote cell cycle arrest and abrogation of parasite motility [19]. In 1992, a new indolocarbazole (ICZ), 7-oxostaurosporine (7OSTS, **2**), exhibited strong human PKC inhibition [20]. One year later, K252c (**8**), the staurosporine aglycone, and other related ICZ aglycones were synthesized and proven to be potent PKC inhibitors [21]. An analogue of **2**, rebeccamycin (**5**), isolated from *Streptomyces aerocolonigenes*, demonstrated no inhibitory effect on PKC, but causes the inhibition of DNA topoisomerase I [22].

Recently, STS (**1**) isolated from cultures of *S. sanyensis* was demonstrated to be active against both cyst and trophozoite forms of *Acantamoeba castellanii* Neff, and induced PCD via the mitochondrial pathway [23]. Despite STS being extensively used for PKC inhibition and its antiparasitic effects having been described, the antikinetoplastic properties against *Leishmania* spp. and *Trypanosoma* sp. of other related ICZs such as 7OSTS (**2**), 4′-demethylamine-4′-oxostaurosporine (4′D4′OSTS, **3**), and streptocarbazole B (SCZ B, **4**) have not been reported, and neither has their structure–activity relationship.

**Figure 1:**
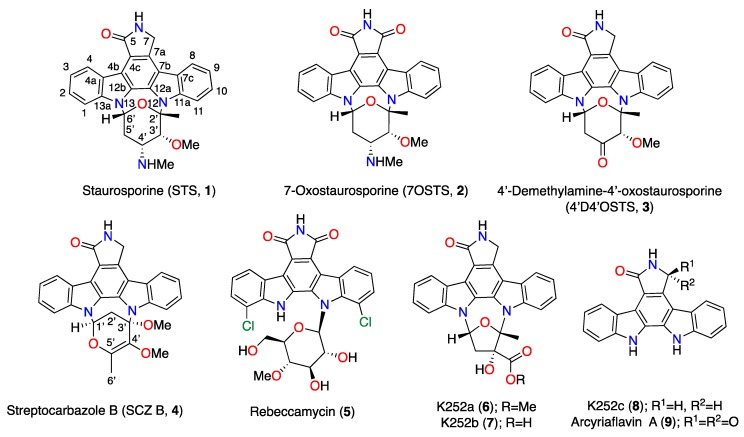
Structures of natural (**1**–**4**) and commercial (**5**–**9**) idolocarbozoles (ICZs).

The aim of this research is to analyze the antikinetoplastid activity of the natural ICZs **2**‒**4** isolated from the *S. sanyensis* PBLC04 strain collected in Ecuador, and to elucidate the mechanism of induced cell death of the most promising molecules compared to the commercial analogues rebeccamycin (**5**), K252a (**6**), K252b (**7**), and their respective aglycones K252c (**8**) and arcyriaflavin A (**9**) (Figure 1) against *Leishmania* spp. and *T*. *cruzi* by confirming the different characteristic events that occur in these protozoa. The antiparasitic drugs in current use have several limitations [4,6,7,8], and therefore new candidate drugs are required.

## 2. Materials and Methods

### 2.1. General Methods

NMR spectra were acquired on a Bruker AVANCE 500 MHz or 600 MHz (Bruker Biospin, Falländen, Switzerland) instrument spectrometer at 300 K) when required. Bruker AVANCE 600 MHz spectrometer is equipped with a 5 mm TCI inverse detection cryoprobe (Bruker Biospin, Falländen, Switzerland). Standard Bruker NMR pulse sequences were utilized. NMR spectra were obtained by dissolving samples in CDCl_3_ (99.9%). EnSpire^®^ Multimode Reader (Perkin Elmer, Waltham, MA, USA) to analyze plates using absorbance values of AlamarBlue^®^ reagent (Bio-Rad Laboratories, Oxford, UK). Thin-layer chromatography (TLC) silica gel plates were used to monitor column chromatography, visualized by UV light (254 nm) and developed with cobalt chloride (2%) as spraying reagent. All reagents and solvents were commercially available and used as received.

### 2.2. Biological Material, Culture and Bioassay-Guided Isolation of Natural ICZ Metabolites ***1***‒***4***

The strain *Streptomyces sanyensis* PBLC04 was isolated from a sediment sample collected in Jambelí mangrove (3°15′792″ S, 80°00′739″ W–03°17′711″ S, 80°01′924″ W), Ecuador. It is included in the microbial collection of Universidad Técnica Particular de Loja (UTPL, Loja-Ecuador). 

*S. sanyensis* PBLC04 was cultured in modified seawater-based medium (A1) (10 g starch, 4 g yeast extract, 2 g proteose peptone, 1 g calcium carbonate, supplemented with 5 mL/L of a solution of potassium bromide (67 mM) and ferric sulfate (20 mM), in 75% seawater) and extracted as previously described [24]. The extract (12.6) g was fractionated by gel filtration on Sephadex LH-20 column (MeOH) to afford four main fractions (SF1-SF4), grouped according to their similar chemical content by TLC. The bioassay analysis of the obtained fractions led us to select the active fractions SF3 and SF4 against *L. amazonensis* with IC_50_ values of 0.43 ± 0.07 µg/mL and 0.08 ± 0.01 µg/mL, respectively. These active fractions were chromatographed using a flash chromatography on an RP18 prepacked cartridge (25–40 µm, 70 g, Götec-Labortechnik GmbH; H_2_O:MeOH, 5mM NH_4_OAc, from 20% to 100% MeOH; 2 mL/min; UV detection at 254 nm), followed by elution on Si-60 open column (230–400 mesh; CHCl_3_:MeOH (9:1)) to yield 65.9 mg of pure staurosporine (**1**) (STS, 65.6 mg, 0.521%); and *n*-Hex:EtOAc:MeOH (2:7:1) to obtain pure 7-oxostaurosporine (**2**) (7OSTS, 1.01 mg, 0.008%), 4′-demethylamino-4′-oxostaurosporine (**3**) (4’D4’OSTS, 1.13 mg, 0.009%), and streptocarbazole B (**4**) (SCZ B, 0.91 mg, 0.007%). The NMR spectra, mass spectrometry and optical rotation data of compounds **1**-**4** have been previously reported [24]. The purity and stability of each compound **1**-**4** was checked by NMR prior to carrying out the biological tests.

### 2.3. Commercial ICZ Analogs ***5***–***9***

ICZ derivatives rebeccamycin (**5**) (CAS no. 93908-02-2), K252a (**6**) (CAS no. 99533-80-9), K252b (7) (CAS no. 99570-78-2), K252c (8) (CAS no. 85753-43-1), and arcyriaflavin A (**9**) (CAS no. 118458-54-1), were all acquired from Cayman Chemical (Ann Arbor, MI, USA) and used as received for bioactivity tests.

### 2.4. Parasite Strain

The activity of compounds **1**-**9** was evaluated against the promastigotes and amastigote stage of *L. amazonensis* (MHOM/BR/77/LTB0016), promastigotes of *L. donovani* (MHOM/IN/90/GE1F8R) and epimastigote of *T. cruzi* (Y strain). Cytotoxicity assays of molecules **1**-**9** were performed against the macrophage J774A.1 cell line, cultured in an RPMI 1640 medium supplemented with 10% fetal bovine serum (FBS) at 37 °C and 5% CO_2_ atmosphere. Promastigotes of both strains of *Leishmania* were cultured in Schneider’s medium (Sigma-Aldrich, Madrid, Spain) supplemented with 10% FBS at 26 °C and were grown to the log phase before performing all the experiments. To carry out the assays, the parasites were cultured in RPMI 1640 medium (Gibco), with or without phenol red. Epimastigotes were cultured in Liver Infusion Tryptose (LIT) medium supplemented with 10% FBS at 26 °C and were grown to the log phase for use in further experiments.

### 2.5. Evaluation of Leishmanicidal, Trypanocidal and Cytotoxic Activities

#### 2.5.1. Leishmanicidal Capacity Assay

The leishmanicidal assay was performed against the promastigote stage of *L. amazonensis* and *L. donovani*. In a sterile 96-well plate, a serial dilution of compounds **1**-**9** was done in RPMI-1640 supplemented with 10% FBS with a final volume of 100µL. Parasites were added to wells to reach a concentration of 10^6^/well. AlamarBlue^®^ at 10% was added into each well and the plate was incubated for 72 h at 26°C [25]. Subsequently, the most active molecules were tested against the intra-macrophages stage of *L. amazonensis*. The anti-amastigote activity was measured according to Jain et al. [26]. Macrophages of the J774A.1 cell line were seeded in a 96-well flat bottom plate at a concentration of 2 × 10^5^/mL in RPMI-1640 supplemented with 10% FBS and was incubated at 37 °C in a 5% CO_2_ environment to allow the almost complete differentiation of the cells. After one hour of incubation, 100 µL of stationary phase promastigotes of 7-day-old culture was added in a 10:1 ratio (2 × 10^6^/mL) and the plates were re-incubated at 37 °C for 24 h to achieve a maximum infection. After the incubation, the wells were washed at least three times to remove the remaining promastigotes and 50 μL of the culture medium (RPMI-1640 with 10% FBS) were added into each well. Separately, and in a 96-deep well plate, a serial dilution of the selected compounds was made with the same medium and then 50 μL of each dilution was added to each well. The plates were incubated at 37 °C, 5% CO_2_ for 24 h. After this incubation, we removed the medium from each well and 30 μL of Schneider medium containing 0.05% SDS was added to each well. The plate was shacked for 30 s and 170 μL of Schneider medium were added to each well. AlamarBlue^®^ at 10% was added into each well and incubated at 26 °C for 72 h. The fluorescence in each well was measured using a spectrofluorimeter at 544 nm excitation, 590 nm emission. Miltefosine (Cayman Chemicals, Vitro SA, Madrid, Spain) was used as reference drug.

#### 2.5.2. Trypanocidal Capacity Assay

The assay was carried out in vitro against epimastigote stage of *T. cruzi*. In a 96-well plate, a serial dilution of compounds **1**‒**9** was incubated for 72 h with the parasite at a concentration of 10^5^ parasite/well. A total of 10% of AlamarBlue^®^ was added to each well and the IC_50_ was calculated. Benznidazole (Sigma-Aldrich, Madrid, Spain) was used as a reference drug.

#### 2.5.3. Cytotoxicity Assay

The cytotoxicity of active compounds was evaluated in J774A.1 macrophage cell line. Serial dilutions of compounds **1**–**9** were plated and incubated with the appropriate cell concentration of macrophages. After 24 h, cell viability was determined using AlamarBlue^®^ method [24]. Miltefosine (Cayman Chemicals, Vitro SA, Madrid, Spain) and benznidazole (Sigma-Aldrich, Madrid, Spain) were used as reference drugs.

### 2.6. Mechanisms of Cell Death

#### 2.6.1. Plasma Membrane Permeability

The SYTOX^®^ Green assay was performed to detect the membrane permeability alterations in parasites. Briefly, 1 × 10^7^ parasites/mL were incubated with the previously calculated IC_90_ for 24 h. SYTOX^®^ Green was added at a final concentration of 1 μM (Molecular Probes). After 15 min of incubation, the increase in fluorescence due to the binding of the dye to the parasitic DNA was observed in an EVOS FL Cell Imaging System AMF4300, Life Technologies, Bothell, WA, USA.

#### 2.6.2. Analysis of Mitochondrial Membrane Potential

The decrease in the mitochondrial membrane potential was detected using a JC-1 Mitochondrial Membrane, Potential Assay Kit, Cayman Chemical. After 24 h of incubation, the previously calculated IC_90_ of the tested molecules, the cells were centrifuged at 1500 rpm for 10min. The pellet was resuspended in JC-1 buffer. After that, 100 µL of each treated culture was added to a black 96-well plate (PerkinElmer) and 10 µL of JC-1 was added, and the plate was incubated for half an hour at 26 °C. Green and red fluorescence intensity was measured using an Enspire microplate reader (PerkinElmer, Massachusetts, USA) for 30 min. In addition, the depolarization of the mitochondrial membrane potential was confirmed by microscopic observation using EVOS FL Cell Imaging System AMF4300, Life Technologies, USA.

#### 2.6.3. Measurement of ATP

ATP level was measured using a Cell Titer-Glo^®^ Luminescent Cell Viability Assay (Promega). The effect of the drug on the ATP production was evaluated by incubating (10^7^ cells/mL) with the previously calculated IC_90_ of the tested molecules for 24 hours. The luminescence was measured using an Enspire microplate reader (PerkinElmer, Waltham, MA, USA).

### 2.7. Statistical Analysis

The half maximal inhibitory concentration (IC_50_) and the cytotoxicity concentration (CC_50%_) were determined by nonlinear regression analysis with 95% confidence limits. All experiments were performed three times, in duplicates for each concentration tested, and the mean values were also calculated. A Tukey test was used for analysis of the data.

## 3. Results 

### 3.1. ICZ Metabolites: Natural Source and Bioassay-Guided Isolation

The strain S. sanyensis PBLC04, isolated from sediment samples collected in Jambelí mangrove, Ecuador, was cultured in 30 L of a seawater-based modified A1 medium. The biomass extract (12.6 g) was chromatographed by gel filtration on Sephadex LH-20 to lead the active fractions SF3 and SF4 against Leishmania amazonensis with IC_50_ values of 0.43 ± 0.08 µg/mL and 0.09 ± 0.004 µg/mL, respectively. As previously described by our research group [24], further chromatographic steps, first by flash chromatography on a RP18 prepacked cartridge, followed by final purification on a Si-60 open column yielded the major compound in the extract, staurosporine (STS) **1** (65.6 mg) [27], and three minor related ICZ metabolites: 7-oxostaurosporine (7OSTS) **2** (1.01 mg) [20], 4′-demethyl-4′-oxostaurosporine (4′D4′OSTS) **3** (1.13 mg) [28], and streptocarbazol B (SCZ B) **4** (0.91 mg) [29]. The spectroscopic data of compounds **1**‒**4** were also reported [24] and compared with those previously described to confirm their structures.

### 3.2. Antiparasitic Assays

#### Antikinetoplastid Activities

Leishmanicidal and trypanocidal activities of natural ICZ compounds **1**–**4** and the structurally related commercial analogues **5**–**9** were determined based on a dose-dependent application against promastigotes of both *L. amazonensis* and *L. donovani* and epimastigotes of *T. cruzi.* The obtained values of concentrations inhibiting 50% (IC_50_) of parasites are summarized in Table 1 and expressed in µM.

Compounds **3** and **4** did not show activity against *L. donovani* at concentrations below 40 μM. Rebeccamycin **5** and the aglycones **8** and **9** were completely inactive against all tested parasites. The natural ICZ metabolites **1** and **2** showed the lowest IC_50_ values, comparable to the reference drug for leishmanicidal (miltefosine IC_50_ = 6.48 ± 0.24 µM) or trypanocidal (benznidazole IC_50_ = 6.94 ± 1.94 µM) treatments.

On the other hand, the toxicity of all compounds was evaluated against the J774A.1 cell line of mouse macrophages as cytotoxic concentration 50 (CC_50_), a concentration in which the population of cells is reduced to 50%. The results are summarized in Table 2 to show the low toxicity of ICZs **3**, **4** and **7** and the aglycones **8**-**9**. The most toxic compounds were rebeccamycin (**5**) and K252a (**6**) with CC_50_ values of 1.42 ± 0.19 µM and 1.07 ± 0.21 µM, respectively.

The effect of the natural ICZs **1**‒**4** on amastigotes of *L. amazonensis* is shown in Table 3. All tested compounds are active with similar IC_50_ values compared to miltefosine, with the exception of 7OSTS (**2**), which is the most potent compound tested among minor metabolites with an IC_50_ of 0.10 ± 0.00 µM. Furthermore, the calculated selectivity index (SI) of **2** is over 2-fold the value obtained for the reference drug to treat leishmaniasis.

### 3.3. Mechanisms of Cell Death

Programmed cell death (PCD) pathways are critical for parasite development and infection, and, consequently, the ability of a molecule to target those mechanisms are considered of relevance in terms of therapeutic potential [31]. The promising results showed by 7OSTS (**2**) prompted us to continue the experimental analysis of its mechanisms of action.

#### 3.3.1. Mitochondrial Damage in *Leishmania amazonensis* Induced by 7-oxostaurosporine (**2**)

**Figure 2:**
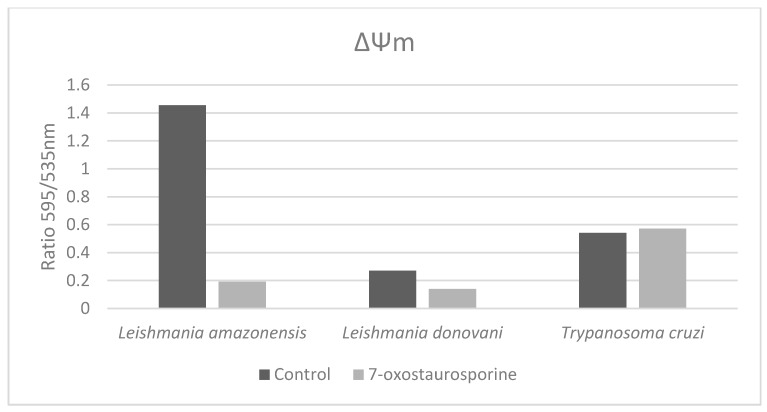
Changes in the mitochondrial membrane potential of *Leishmania* spp. promastigotes and *T. cruzi* epimastigotes after treatment at the IC_90_ of 7OSTS (**2**) for 24 h. Control corresponds to untreated parasites.

**Figure 3:**
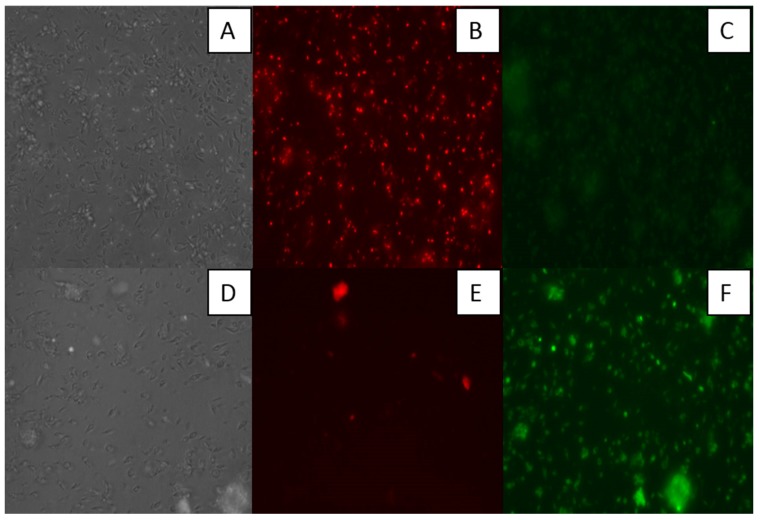
Effect of 7OSTS (**2**) on the mitochondrial potential in cells of *L. amazonensis* treated with the IC_90_ for 24 h (**D**), (**E**) and (**F**). Negative control are non-compound-treated cells of *L. amazonensis* (**A**), (**B**) and (**C**). Due to a collapse in mitochondrial potential, the JC-1 dye in dimers (red) disappeared from mitochondria (**E**) and remained in the cytoplasm in its monomeric form, green fluorescence (**F**). (**A**), (**D**): visible light. (**B**), (**E**): (Ex: 531/40 Em: 593/40) Excitation/Emission (nm) for Red Fluorescent Protein (RFP). (**C**), (**F**): (Ex: 470/22 Em: 525/50) for Green Fluorescent Protein (GFP). Images taken by EVOS FL inverted microscope (Invitrogen) (40X).

The effect of 7OSTS (**2**) on the mitochondrial membrane potential was measured in promastigotes of *L. amazonensis* and *L. donovani*, and *T. cruzi* epimastigotes. We could observe an intense effect of the mitochondrial membrane potential (ΔΨm), when *L. amazonensis* promastigotes were treated with **2** at the IC_90_ concentration (8.36 µM) (Figure 2 and Figure 3). The IC_90_ value was used to increase the population of affected parasites and to reduce the experimental time. The presence of JC-1 dye in the cytoplasm in its monomeric form (green fluorescence) confirms the depolarization of *L. amazonensis* mitochondrial membrane (Figure 3). In contrast, we did not observe any change in *L. donovani* promastigotes or *T. cruzi* epimastigotes treated with the IC_90_ of the 7OSTS (**2**) (Figure 2).

#### 3.3.2. Cytoplasmic Membrane Permeability in *Leishmania donovani* and *Trypanosoma cruzi* Induced by 7-oxostaurosporine (**2**)

**Figure 4:**
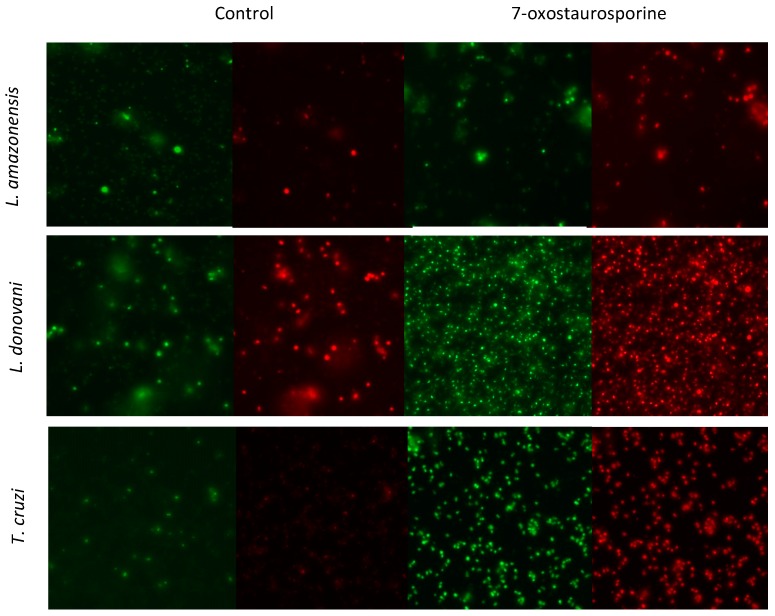
SYTOX Green (green) and propidium iodide (red) stainings in control and treatment with 7OSTS (**2**) on the three different strains after 24 h. Images taken by EVOS FL inverted microscope (Invitrogen) (40X).

The cytoplasmic membrane permeability of *L. amazonensis, L. donovani* and *T. cruzi* after 24 h treatment with the IC_90_ of 7OSTS (**2**) by the SYTOX Green assay, reveals a remarkable membrane alteration in cultures of *L. donovani* and *T. cruzi*, as shown in Figure 4. Similarly, the same effect is also observed in death cells by propidium iodide staining. Interestingly, the cytoplasmic membrane of *L. amazonensis* does not seem to be permeable under the experimental conditions. 

## 4. Discussion

The family of ICZs have been the focus of intense research as chemotherapeutics, and some of them have advanced into clinical trials [32,33]. According to the proteins they target, they have been subdivided into two broad groups. One is represented by STS (**1**) and includes the ICZ compounds that are potent inhibitors of protein kinases (PKC, PKA, CDK2, etc.), whereas the second group, modified in the sugar moiety such as rebeccamycin (**5**), are potent stabilizers of DNA topoisomerase-I [33,34,35,36].

**Figure 5:**
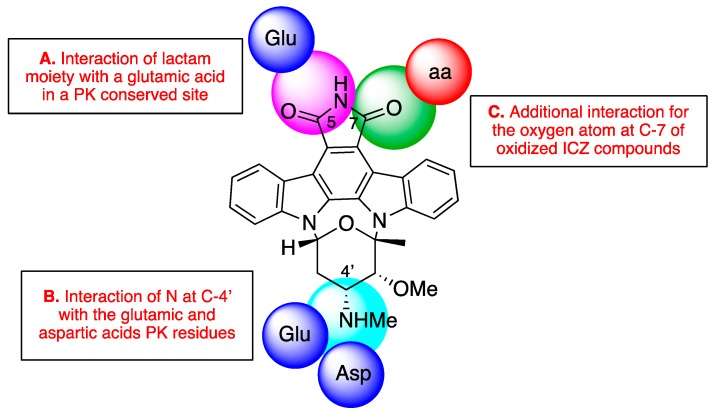
Interaction model for staurosporine (STS)-related compounds with protein kinases (PKs) [33,34,35,36]. (**A**), (**B**) and (**C**) describe the key-interactions with conserved residues at the active site.

The initials studies on the STS-PK complex used PKA and CDK2 kinase models [34]. These studies and those completed later revealed a critical hydrogen bond interaction between the heteroatoms of the lactam moiety of STS with a conserved glutamic residue at the protein active site (Figure 5A). Moreover, in closely STS-related compounds, the methyl amine group at C-4′ is involved in the formation of two hydrogen bonds with amino acids involved in the catalytic pocket, such as Glu and Asp (Figure 5B). These interactions fix a boat-type conformation of the sugar moiety, which is perpendicularly located to the planar sp^2^ ICZ fragment. This specific conformation has been related with the inhibitory activity of protein kinases. Thus, ICZs functionalized at the carbon C-4′ show an increased activity in the function of the number of hydrogen bonds between the nitrogen at the methyl amino moiety of neighboring protein residues (Figure 5B) [34,35].

Some protein kinases form an additional hydrogen bond with the oxygen atom present at carbon C-7 in oxidized ICZs compounds (Figure 5C). In these cases, water molecules are involved in the coordination. Therefore, it appears to be a differentiating element that may cause a reinforcement in the interaction of oxidized derivatives at C-7 with PKs and, consequently, increased activity. One example is UCN-01 (7-hydroxystaurosporine), which shows similar inhibition profiles to STS with eleven kinases. Five of those kinases have a residue equivalent to Thr222 in PDK1; and PKB and PKC have Thr at the Val143 position of PDK1, thus all may have formed an additional hydrogen bond to the 7-hydroxy group [33].

Based on this interaction model, in the present study the antiprotozoal activities of the natural compounds **1**-**4** beside the commercial ICZs rebeccamyccin (**5**), K252a (**6**), K252b (**7**), K252c (**8**), and arcyriaflavin A (**9**) have been analyzed in order to establish a plausive approach to the mode of action and to provide a preliminary structure–activity relationship (SAR). Thus, the DNA topoisomerase-I inhibitor rebeccamycin (**5**) showed no activity (IC_50_ > 40 µM) against all tested parasites, suggesting that the most probable inhibition mechanism for natural compounds **1**‒**4** affects parasite PKs. Similarly, the aglycones of STS (**1**) and 7OSTS (**2**), K252c (**8**) and arcyriaflavin A (**9**), respectively, were inactive at concentrations below 40 µM, and confirm the relevance of the sugar moiety in the inhibition of parasite PKs.

**Figure 6:**
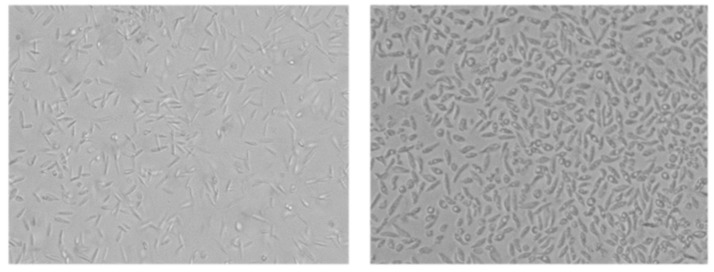
Left: control of non-treated promastigotes of *L. donovani*; right: promastigotes of *L. donovani* treated with 7OSTS (**2**) at 10 µM. Images taken by EVOS FL inverted microscope (Invitrogen) (20X).

The most active STS-related compound was 7OSTS (**2**), which showed IC_50_ values of 3.58 ± 1.10; 0.56 ± 0.06 and 1.58 ± 0.52 µM against *L. amazonensis; L. donovani* and *T. cruzi,* respectively (Table 1), which are slightly improved with respect to those of STS (**1**)-treated *L. donovani* and *T. cruzi*, and could be due to the presence of a carbonyl group at C-7 in **2**. Of note is the Selectivity Index (CC_50_/IC_50_) of 7OSTS **2** against amastigotes of *L. amazonensis* (Table 3) when compared with murine macrophages J774A.1, which improves that of miltefosine. Thus, when *L. donovani* is treated with **2** at 10 µM, parasites suffer morphological changes compared to control cells, with differences in the size and appearance of the flagellar pocket (increased with treatment), and invagination of the plasma membrane in the place where the flagellar system is assembled to the body of the parasite (Figure 6), characteristic damages for PKs inhibition [9,19,37,38].

Furthermore, ICZ analogues, K252a (**6**) and K252b (**7**), first isolated from the actinomycete *Nocardiopsis* [39] differ from STS (**1**) in the sugar moiety (Figure 1 and Figure 5). Whereas **6** is a reversible cell-permeable inhibitor of phosphorylase kinase (IC_50_ = 1.7 nM), protein kinase A (PKA) (IC_50_ = 140 nM), and protein kinase C (PKC) (IC_50_ = 470 nM) [40,41], **7** is used as a non-permeable PKC inhibitor [42,43,44]. The antikinetoplastid screening show a similar behavior of *L. amazonensis* when treated with K252a (**6**) and 7OSTS (**2**), whereas the effectiveness of K252b (**7**) is lower (IC_50_ = 20.62 ± 4.50 µM), indicating that the most probable mechanism of action of **2** affects intracellular PKs of *L. amazonensis.* On the contrary, *L. donovani* and *T. cruzi* responded in a similar way when treated with **6** and **7**. 

In summary, 7OSTS (**2**) possesses potent activity against all three tested species, similar to that showed by STS (**1**). These results could be explained based on the fact that, structurally, both compounds possess the lactam group and the methyl amine at the C-4’ position, with similar orientation and conformation, and thus, **1** and **2** interact with the conserved aminoacidic residues in parasite PKs. In addition, the differences found between STS (**1**) and 7OSTS (**2**) could be justified based on the positive or negative interaction with the active core of the target parasite PKs, due to the additional functionalization at C-7 position (Figure 5C). For the rest of tested substances, **3**-**9**, the crucial interactions between the N-Me moiety at the C-4′ position with the active center of the PK cannot be produced, and therefore their activity is lower than those compounds that contain the methyl amino fragment.

## 5. Conclusions

We have found a clear correlation between the antikinetoplastid activities observed and the structural elements of the studied ICZs. Both STS (**1**) and 7OSTS (**2**) possess potent activities against all three tested species. Their similar structural features, orientation and conformation assure the interaction with conserved aminoacidic residues of the PKs of parasites. Among all tested compounds, 7OSTS (**2**) was also revealed to be particularly selective against the amastigote stage of *L. amazonensis,* and new studies should be oriented to explore the therapeutic potential and mode of action of this molecule in order to develop new antileishmanial compounds.

## Figures and Tables

**Table 1:** Antikinetoplastid activity of ICZ metabolites isolated from *Streptomyces sanyensis* (**1**‒**4**) and commercial ICZs (**5**‒**9**) against *Leishmania* and *Trypanosoma* species. IC_50_ values are reported in µM concentrations (Mean concentration ± SD).

Compounds	*L. amazonensis* IC_50_ (µM)	*L. donovani* IC_50_ (µM)	*T. cruzi* IC_50_ (µM)
**1** STS	0.08 ± 0.02	2.07 ± 0.14	3.63 ± 0.77
**2** 7OSTS	3.58 ± 1.10	0.56 ± 0.06	1.58 ± 0.52
**3** 4′D4′OSTS	17.10 ± 4.78	> 40	17.10 ± 1.64
**4** SCZ B	10.44 ± 0.21	> 40	12.50 ± 2.06
**5** Rebeccamycin	> 40	> 40	> 40
**6** K252a	5.90 ± 0.96	8.09 ± 1.12	4.00 ± 0.24
**7** K252b	20.62 ± 4.50	4.45 ± 0.71	7.41 ± 0.93
**8** K252c	> 40	> 40	> 40
**9** Arcyriaflavin A	> 40	> 40	> 40
Miltefosine *	6.48 ± 0.24	3.32 ± 0.27	-
Benznidazole *	-	-	6.94 ± 1.94

* Reference compounds.

**Table 2:** Toxicity against murine macrophage J774A.1 (CC_50_) measured by AlamarBlue assay. CC_50_ are reported in µM concentrations. (Mean concentration ± SD).

Compounds	Macrophage J774A.1 CC_50_ (µM)
**1** STS	8.74 ± 0.72
**2** 7OSTS	5.20 ± 1.75
**3** 4′D4′OSTS	> 40
**4** SCZ B	> 40
**5** Rebeccamycin	1.42 ± 0.19
**6** K252a	1.07 ± 0.21
**7** K252b	> 40
**8** K252c	35.4 ± 2.47
**9** Arcyriaflavin A	> 40
Miltefosine *	72.19 ± 3.06
Benznidazole *	400.00 ± 4.00

* Reference compounds.

**Table 3:** Leishmanicidal effect of ICZs **1**‒**4** against the intracellular stage (amastigotes), and its comparison with the reference drug by the selectivity index (CC_50_/IC_50_).

Compounds	*L. Amazonensis* Amastigotes IC_50_ (µM)	Selectivity Index (CC_50_/IC_50_)
**1** STS	10 *	--
**2** 7OSTS	0.10 ± 0.00	52
**3** 4′D4′OSTS	2.03 ± 0.27	20
**4** SCZ B	2.47 ± 0.09	16
Miltefosine **	3.12 ± 0,30	23

* Not tested in this assay. Data from Becker et al., 1997 [30]; ** Reference compound.
